# Metagenomic next-generation sequencing for lung cancer low respiratory tract infections diagnosis and characterizing microbiome features

**DOI:** 10.3389/fcimb.2024.1518199

**Published:** 2025-01-23

**Authors:** Yao Liu, Bohan Yang, Qi Qi, Shijie Liu, Yiheng Du, Linlin Ye, Qiong Zhou

**Affiliations:** Department of Respiratory and Critical Care Medicine, Union Hospital, Tongji Medical College, Huazhong University of Science and Technology, Wuhan, China

**Keywords:** metagenomic next-generation sequencing (mNGS), lung cancer, lower respiratory tract infections (LRTIs), microbiome, gene set variation analysis (GSVA)

## Abstract

**Background:**

The capability of mNGS in diagnosing suspected LRTIs and characterizing the respiratory microbiome in lung cancer patients requires further evaluation.

**Methods:**

This study evaluated mNGS diagnostic performance and utilized background microbial sequences to characterize LRT microbiome in these patients. GSVA was used to analyze the potential functions of identified genera.

**Results:**

Bacteria were the most common pathogens (n=74) in LRTIs of lung cancer patients, and polymicrobial infections predominated compared to monomicrobial infections (p<0.001). In diagnosing LRTIs in lung cancer patients, the pathogen detection rate of mNGS (83.3%, 70/84) was significantly higher than that of sputum culture (34.5%, 29/84) (p<0.001). This result was consistent with that of non-lung cancer patients (p<0.001). Furthermore, in the specific detection of bacteria (95.7% vs. 22.6%) and fungi (96.0% vs. 22.2%), the detection rate of mNGS was also significantly higher than that of CMTs mainly based on culture (p<0.001, p<0.001). However, in the detection of CMV/EBV viruses, there was no significant difference between the detection rate of mNGS and that of viral DNA quantification (p = 1.000 and 0.152). mNGS analysis revealed *Prevotella*, *Streptococcus*, *Veillonella*, *Rothia*, and *Capnocytophaga* as the most prevalent genera in the LRT of lung cancer patients. GSVA revealed significant correlations between these genera and tumor metabolic pathways as well as various signaling pathways including PI3K, Hippo, and p53.

**Conclusion:**

mNGS showed a higher pathogen detection rate than culture-based CMTs in lung cancer patients with LRTIs, and also characterizing LRT microbiome composition and revealing potential microbial functions linked to lung carcinogenesis.

## Introduction

Lung cancer patients are susceptible to lower respiratory tract infections (LRTIs) due to their immunocompromised status (Peng et al., 2021). However, etiological diagnosis of LRTIs remains challenging, with causative pathogens unidentified in ~50% of cases (Zhu et al., 2018), especially among lung cancer patients, whose infections often present with polymicrobial etiology (Peng et al., 2021; Sun et al., 2021). Moreover, the inability of lung cancer patients to produce adequate sputum or tolerate invasive procedures such as bronchoalveolar lavage fluid (BALF) further limited our ability to identify the infecting pathogens (Rolston et al., 2007). Conventional microbiological tests (CMTs), including microbial culture, antibody-antigen assays, and nucleic acid testing, are widely used for the etiological diagnosis of LRTIs (Han et al., 2023). However, these methods have significant limitations. Standard microbial cultures grow slowly and are affected by stringent environmental conditions and low sensitivity (Gadsby et al., 2019; Liu et al., 2021a). Additionally, culture results can be influenced by antimicrobial agents and the quality of sample collection (Datta et al., 2017). While tests for specific pathogens, such as pneumococcal urinary antigen and respiratory virus nucleic acid detection, can help identify suspected pathogens or differentiate between bacterial and viral infections, they are often time-consuming and prone to high rates of missed diagnoses (Murphy et al., 2020). Furthermore, given that many hospitals lack complex detection methods for specialized pathogens, such as immunofluorescence microscopy for *Pneumocystis jirovecii*, using comprehensive molecular biology techniques becomes essential for aiding in the etiological diagnosis of LRTIs in lung cancer patients (Liu et al., 2021). Metagenomic Next-Generation Sequencing (mNGS) is a high-throughput nucleic acid sequencing technique that enables unbiased identification of diverse pathogenic microbes, including viruses, bacteria, fungi, and parasites, within clinical specimens, and mNGS has seen widespread adoption in the etiological diagnosis of infectious diseases, especially for the detection of rare, novel, and complicated infection diseases (Chiu and Miller, 2019; Li et al., 2021). Previous studies compared the diagnostic capabilities of mNGS and CMTs in the diagnosis of LRTIs among immunocompromised patients, such as those admitted to the intensive care unit, with rheumatic diseases, and with malignancies (Peng et al., 2021; Shi et al., 2022; Sun et al., 2022; Wang et al., 2024). However, considering the diversity of pathogen profiles among immunocompromised patients with various underlying conditions that could impact diagnostic performance, it becomes imperative to focus on analyzing studies specifically related to a cohort of patients with lung cancers.

In addition, mNGS enables the sequencing and analysis of mixed microbial genomes (Schloss and Handelsman, 2003), allowing for the identification of not only pathogenic bacteria but also the characterization of the lower respiratory tract (LRT) microbiome using background flora data in lung cancer patients. Accumulating evidence suggests a strong link between LRT microbiome dysbiosis and lung carcinogenesis, potentially through mechanisms such as upregulation of host inflammatory pathways, activation of oncogenic molecular pathways, and release of carcinogenic microbial metabolites (Gustafson et al., 2010; Ramírez-Labrada et al., 2020; Sepich-Poore et al., 2021). Importantly, LRT microbiome dysbiosis can also modulate host immune responses, ultimately impacting the effectiveness of anti-tumor treatment. Routy and Derosa et al. demonstrated reduced efficacy of immune checkpoint inhibitors (ICIs) therapy in patients with advanced epithelial cancers, including lung cancer, who received antibiotics (Derosa et al., 2018; Routy et al., 2018).

Given these challenges, prompt pathogen identification in LRTIs of lung cancer patients is crucial, facilitating optimal treatment selection and improving prognosis. Additionally, to further elucidate the role of the LRT microbiome in disease development, this study investigated its composition and function in lung cancer patients.

## Methods

### Study design and patient population

This retrospectively enrolled patients with suspected LRTIs who visited Wuhan Union Hospital from March 2021 to January 2023. Patients meeting any one of the following criteria from 1-4 and criteria 5-7 were included in this study (Mandell et al., 2007): (1) New-onset cough, sputum, or worsening of pre-existing respiratory disease symptoms, with or without purulent sputum, chest pain, dyspnea, and hemoptysis; (2) fever; (3) signs of pulmonary consolidation or moist rales on auscultation; (4) peripheral blood leukocyte count higher than 10×10^9^/L or lower than 4×10^9^/L; (5) lung imaging showing new patchy infiltrates, lobar or segmental consolidation, ground-glass opacities, or interstitial changes, with or without pleural effusion; (6) patients with a history of primary lung cancer; (7) patients who underwent mNGS. Patients without complete medical records and those who died within 24 hours after mNGS detection were excluded. A total of 110 patients were ultimately included in this study, which has been approved by the Wuhan Union Hospital (No. 2024-0158-01). Due to its retrospective design and the use of anonymized data, the ethics committee waived the requirement for informed consent. We confirm that all procedures were conducted in compliance with the relevant guidelines and regulations.

Propensity score matching (PSM) was used to control potential confounding bias in this study. Using the nearest neighbor matching algorithm from the MatchIt package, propensity scores were calculated through a generalized linear model with logistic regression. The lung cancer diagnosis served as the dependent variable, with covariates including sex, age, application of antibiotics before mNGS, and application of glucocorticoids before mNGS. A caliper width of 0.02 was applied to ensure matching of individuals with propensity scores within this narrow range, thereby enhancing matching precision. Ultimately, we successfully matched 102 lung cancer patients with 102 non-lung cancer patients as controls, all with LRTIs, for subsequent analysis.

### Clinical data collection

This study gathered baseline characteristics and clinical data from patients, encompassing pulmonary function results, imaging features, laboratory tests, antimicrobial treatment, and clinical outcomes. In addition, all patients included in the study contributed pertinent clinical samples, such as BALF, sputum, blood, throat swabs, or urine, for various CMTs, including culture, immunological assays (e.g. antigen detection, (1/3)-β-D-glucans), molecular biology techniques (e.g. multiplex PCR, X-pert MTB/RIF), and direct microscopic examination.

### mNGS of BALF

All patients received a standardized bronchoscopic examination conducted by experienced specialists at our institution. Following the identification of imaging and endoscopic findings, BALF samples were collected from the affected lung regions using sterile saline solution. The BALF samples were promptly cryopreserved and submitted for mNGS analysis. BALF samples underwent comprehensive mNGS analysis by a specialized testing company. The workflow commenced with nucleic acid extraction, followed by library construction. During library preparation, nucleic acids underwent fragmentation and end-repair processes. A single-base adenine (A) was added to the 3’ end of DNA fragments, and adapters (indexes) were ligated to both ends of the DNA fragments using “T-A” ligation, rendering the library compatible with the Illumina sequencing platform. After library quantification and pooling, the prepared sample was hybridized on the sequencing chip and analyzed by next-generation sequencing. Bioinformatics analysis compared detected microbial nucleic acid sequences with comprehensive reference databases, enabling precise identification of microbial species and their relative abundance. The report characterized both pathogenic and commensal microbiota across human body sites, with a detection scope including 10,989 bacterial species (comprising 196 actinobacteria and 159 mycoplasma/chlamydia/rickettsia), 5,050 viral species, 1,179 fungal species, 282 parasitic species. Various sequencing platform parameters were employed to ensure data quality and reliability, including mapping read number (at species and genus levels), abundance (at species levels), depth and coverage rate were critically utilized to eliminate potential interfering microorganisms and generate a definitive official report.

### Infection diagnosis and mNGS result analysis

The final diagnosis was determined through independent review of all patient records by two professional pulmonologists (LY and QQ). The diagnosis of infectious and further identification of pathogenic microorganisms in infectious diseases, were based on clinical manifestations, laboratory findings, chest imaging, microbiological examinations (including CMTs and mNGS), and response to antimicrobial therapy. Disagreements were resolved through discussion or consultation with a senior pulmonologist (YBH) when necessary.

Consistency between mNGS results and clinical diagnosis was defined as either mNGS-positive results matching the final diagnosis or mNGS-negative results with a non-infectious final diagnosis. Inconsistency was defined as either mNGS-positive results discordant with the final diagnosis or mNGS-negative results in cases of infectious disease.

### Gene set variation analysis

Tumor microbiome count data from NSCLC in The Cancer Genome Atlas (TCGA), reported by Narunsky-Haziza et al. (2022) (https://github.com/knightlab-analyses/mycobiome), were used for GSVA. Pathway gene sets were obtained from the Kyoto Encyclopedia of Genes and Genomes (KEGG) database. Spearman’s correlation coefficient was used to assess the relationship between intratumoral microbiota and KEGG pathway in paired TCGA samples. Before GSVA, we applied Haziza’s pipeline to address batch effects in TCGA tumor microbiome data, using Voom to convert taxonomic counts to log-counts per million (log-cpm) and performing supervised normalization (SNM).

### Statistical analysis

Categorical variables were expressed as n (%), while continuous variables were presented as median (25th-75th percentiles). Fisher’s exact test was used for bivariate analysis of discrete variables. All statistical analyses were performed using the Statistical Package for the Social Sciences soft-ware (SPSS) version 25.0 software package (IBM, USA). Two-tailed P values < 0.05 were considered statistically significant. Graphs were generated using GraphPad Prism (version 10). GSVA and PSM were performed using R software (version 4.3.1).

## Results

### Patient demographics

This study included 110 lung cancer patients with suspected LRTIs who underwent mNGS testing at Wuhan Union Hospital between March 2021 and December 2023 (**Table 1**). The cohort was mostly male (87, 79.1%), with median age of 62 years (57.8, 68.3) and body mass index (BMI) of 22.6 (20.5, 24.8). Among all patients, non-small cell lung cancer (NSCLC) was most common (103, 93.6%), mainly comprising lung adenocarcinoma (LUAD) (52, 47.3%) and lung squamous cell carcinoma (LUSC) (38, 34.5%), with the remainder being SCLC (7, 6.4%). In terms of Tumor-Node-Metastasis (TNM)-based classification, the majority of patients (80, 72.7%) were at stage III-IV, whereas a smaller proportion (19, 17.3%) were at stage I-II. The remaining patients (11, 10%) had unknown or unclassified stages. The median hospital stay was 11.5 days (8, 17.8) and mNGS testing was performed a median of 4 days (3, 7) after admission. Additionally, the median duration between the last anti-tumor treatment and mNGS testing was 20 days (0, 61.5). Prior to mNGS testing, a majority of (95, 86.4%) patients received antibiotic therapy. A subset of (20, 18.2%) patients were treated in the intensive care unit (ICU), of whom a small number (8, 7.3%) ultimately died. Among the 110 lung cancer patients, 102 were 1:1 matched with non-lung cancer patients based on PSM. Standardized mean differences (SMD) for covariates were <0.01, indicating optimal covariate balance (**Supplementary Table 1**).

**Table 1:** Demographic and baseline characteristics of 110 lung cancer patients with suspected LRTIs.

Characteristic	Value
Age, year, median (IQR)	62 (57.8, 68.3)
Male, n (%)	87 (79.1%)
BMI,median (IQR)	22.6 (20.5, 24.8)
Infection	
Yes	96 (87.3%)
No	14 (12.7%)
Imaging fingdings	
Unilateral	57 (51.8%)
Bilateral	53 (48.2%)
Infection site matches primary lung cancer location	
Yes	87 (79.1%)
No	23 (20.9%)
Application of antibiotics before mNGS, n (%)	95 (86.4%)
Antibacterial therapy	93 (84.5%)
Antibacterial-antifungal therapy	15 (13.7%)
Antibacterial-antiviral therapy	11 (10.0%)
Antibacterial-antifungal-antiviral therapy	3 (2.7%)
Histology	
NSCLC	103 (93.6%)
LUAD	52 (47.3%)
LUSC	38 (34.5%)
Others	13 (11.8%)
SCLC	7 (6.4%)
cTNM stage	
I-II	19 (17.3%)
III-IV	80 (72.7%)
Hospital LOS (days), median (IQR)	11.5 (8.0, 17.8)
Duration between admission and mNGS (day), median (IQR)	4 (3,7)
Duration between the last anti-tumor treatment and mNGS (day), median (IQR)	20 (0, 61.5)
ICU admission rate, n (%)	20 (18.2%)
Mortaily, n (%)	8 (7.3%)

LRTIs, Lower respiratory tract infections; IQR, interquartile range; BMI, body mass index; mNGS, metagenomic next-generation sequencing; NSCLC, non-small cell lung cancer; LUAD, lung adenocarcinoma; LUSC, lung squamous cell carcinoma; SCLC, small cell lung cancer; cTNM, tumor-node-metastasis; LOS, length of stay; ICU, intensive care unit.

### LRTIs features in lung cancer patients

**Figure 1:**
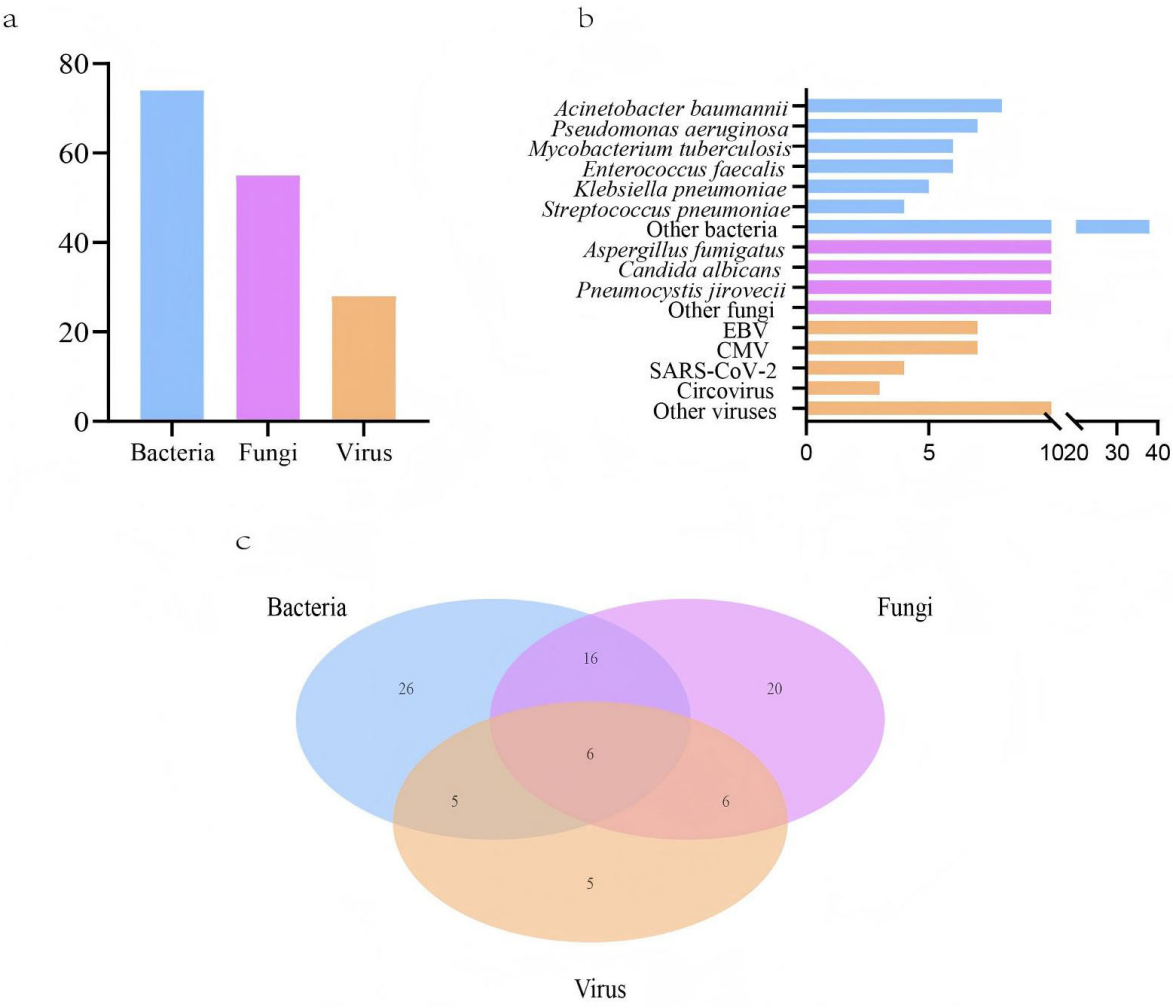
Pathogens detected in lower respiratory tract infections (LRTIs) of lung cancer patients (n=110) by metagenomic next-generation sequencing (mNGS) and conventional microbiological tests (CMTs). **(A)** Composition of pathogens in lung cancer patients with positive mNGS and CMTs results; **(B)** Distribution of pathogens identified by mNGS and CMTs; **(C)** The number of lung cancer patients with LRTIs for various pathogens.

LRTIs were diagnosed in 96 lung cancer patients (96, 87.3%), while 14 patients (12.7%) were attributed to non-infectious etiologies, including disease progression and immune-related pneumonitis (**Table 1**). Among the 96 patients, 84 had microbiologically confirmed diagnoses, yielding 74 bacterial, 55 fungal, and 28 viral strains (**Figure 1A**). The most common bacterial infections were *Acinetobacter baumannii* (8, 5.1%), *Pseudomonas aeruginosa* (7, 4.5%), and *Mycobacterium tuberculosis* (6, 3.8%) infections. For fungal infections, *Aspergillus fumigatus* (17, 10.8%), *Candida albicans* (14, 8.9%), and *Pneumocystis jirovecii* (14, 8.9%) infections were most prevalent. Viral infections were predominantly caused by Epstein-Barr virus (EBV) (7, 4.5%) and cytomegalovirus (CMV) (7, 4.5%) (**Figure 1B**). Among infected lung cancer patients, bacterial infections were most prevalent (53, 55.2%), followed by fungal (48, 50.0%) and viral (22, 22.9%) infections. In cases of polymicrobial infections, bacterial-fungal infections were most common (16, 16.7%), while bacterial-fungal-viral infections were observed in a small subset of patients (6, 6.3%) (**Figure 1C**). Overall, in LRTIs among lung cancer patients, polymicrobial infections were characterized by a significantly higher prevalence of bacterial (p<0.001), fungal (p<0.001), and viral infections (p<0.001), particularly *Pneumocystis jirovecii*, CMV (p=0.023), and EBV (p<0.001) infections (**Table 2**).

**Table 2:** LRTIs pathogens in 110 lung cancer patients.

	All (n=84)	Monomicrobial (n=39)	Polymicrobial (n=45)	*P-*value
Bacteria	74	16	58	<0.001
*Acinetobacter baumannii*	8	2	6	0.131
*Pseudomonas aeruginosa*	7	3	4	1.000
*Mycobacterium tuberculosis*	6	2	4	0.567
*Enterococcus faecalis*	6	1	5	0.080
Other bacteria	47	8	39	<0.001
Fungi	55	16	39	<0.001
*Aspergillus fumigatus*	17	6	11	0.169
*Candida albicans*	14	7	7	1.000
*Pneumocystis jirovecii*	14	1	13	<0.001
Other fungi	10	2	8	0.023
Viruses	28	4	24	<0.001
Cytomegalovirus	7	1	6	0.029
Epstein-Barr Virus	7	0	7	<0.001
Other viruses	14	3	11	0.007

All pathogens defined in this table are all final diagnoses.

### Assessment of mNGS diagnostic performance in lung cancer patients

**Figure 2:**
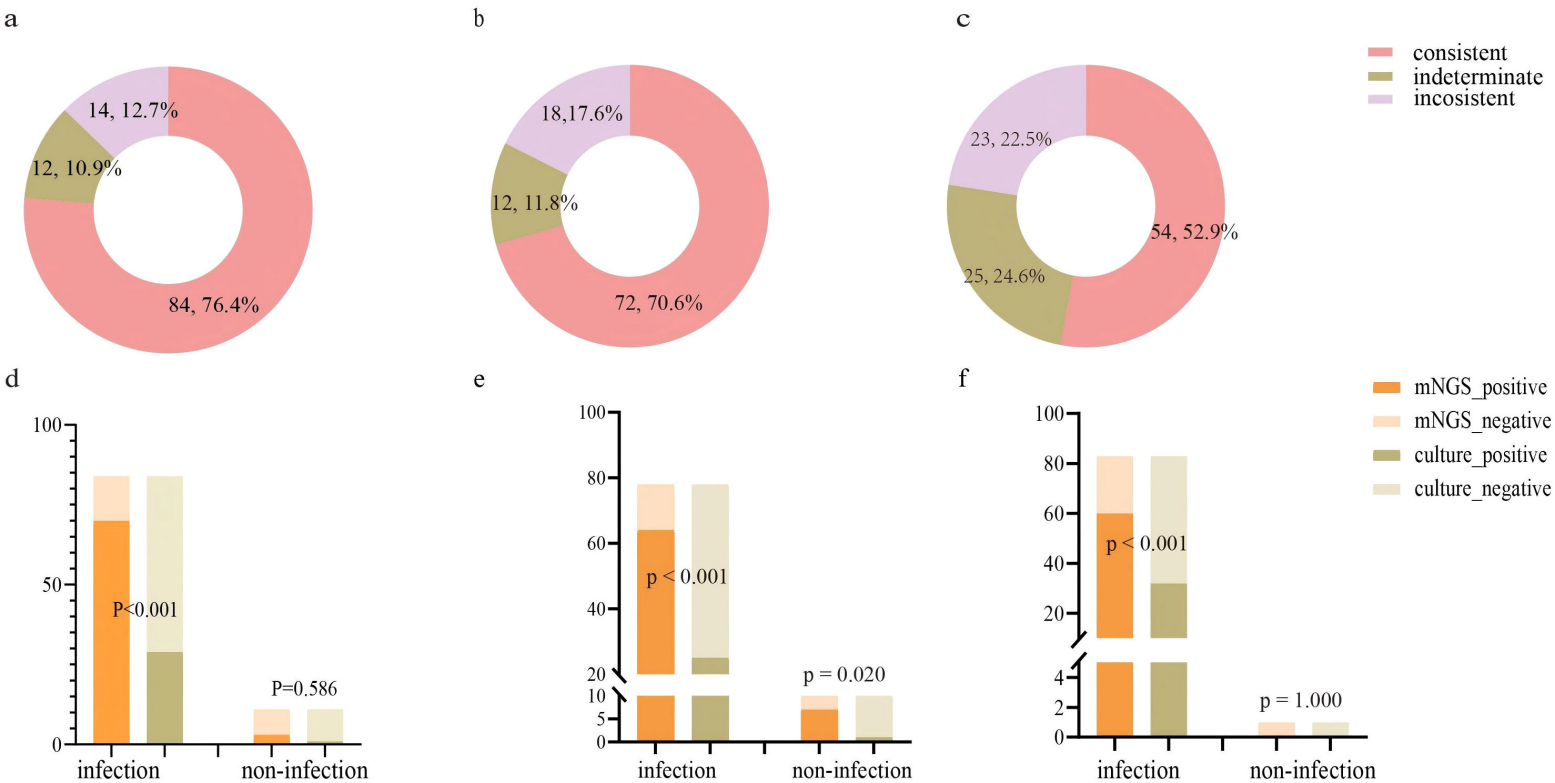
Evaluating the performance of mNGS in diagnosing LRTIs in lung cancer patients and non-lung cancer patients. **(A)** Consistency analysis between mNGS and final diagnosis in lung cancer patients (n=110); **(B)** Consistency analysis between mNGS and final diagnosis in matched lung cancer patients (n=102); **(C)** Consistency analysis between mNGS and final diagnosis in matched non-lung cancer patients (n=102); **(D)** The detection performance of mNGS and sputum culture in lung cancer patients (n=95). **(E)** The detection performance of mNGS and sputum culture in matched lung cancer patients (n=88). **(F)** The detection performance of mNGS and sputum culture in matched non-lung cancer patients (n=84).

This study further evaluated the diagnostic capability of mNGS. We initially assessed the consistency between mNGS results and clinical diagnoses. The analysis revealed 84 (76.4%) cases with consistent mNGS and clinical diagnoses, 14 (12.7%) cases with inconsistent results, and 12 (10.9%) indeterminate cases (**Figure 2A**). About 86.4% (86.4%, 95/110) patients underwent sputum culture. The diagnostic accuracy between mNGS and sputum culture was further compared in these patients (**Figure 2D**; **Supplementary Table 2**). Among patients diagnosed with infection, the pathogens detection rate was higher in mNGS (83.3%, 70/84) than the sputum culture (34.5%, 29/84) (p<0.001). In non-infected patients, no significant difference was observed between the mNGS (72.7%, 8/11) and the sputum culture (90.9%, 10/11) (p=0.586). Additionally, we examined the differences in mNGS result consistency with clinical diagnoses between matched lung cancer and non-lung cancer patients. In 102 propensity score-matched lung cancer patients, 72 (70.6%) cases showed consistent mNGS and clinical diagnoses, 18 (17.6%) cases were inconsistent, and 12 (11.8%) cases were indeterminate (**Figure 2B**). Among 88 lung cancer patients tested with both mNGS and sputum culture, mNGS demonstrated significantly higher pathogen detection rates compared to sputum culture in both infected (82.1% vs. 32.1%, p<0.001) and non-infected patients (70.0% vs. 10.0%, p=0.020) (**Figure 2E**; **Supplementary Table 2**). Similarly, in 102 matched non-lung cancer patients, mNGS revealed 54 (52.9%) consistent, 23 (22.5%) inconsistent, and 25 (24.6%) indeterminate cases (**Figure 2C**). Of 84 non-lung cancer patients simultaneously tested, mNGS showed significantly higher pathogen detection in infected patients (72.3% vs. 38.5%, p<0.001), with comparable results in non-infected patients (0.0%, 0/1) (**Figure 2F**; **Supplementary Table 2**). Finally, we compared mNGS and CMTs mainly including culture in diagnosing diverse pathogens across matched lung cancer and non-lung cancer patients to evaluate mNGS’s pathogen-specific detection capability. The results showed that in lung cancer and non-lung cancer patients, mNGS demonstrated higher positive detection rates for bacteria (p<0.001, p<0.001) and fungi (p<0.001, p=0.006) compared to CMTs (**Table 3**). Moreover, comparing the performance of mNGS in BALF and EBV-DNA/CMV-DNA testing in whole blood and plasma for diagnosing EBV and CMV infections revealed no significant differences between lung cancer and non-lung cancer patients (**Supplementary Table 3**).

**Table 3:** Diagnostic performance of CMTs and mNGS in matched lung cancer patients and non-lung cancer patients with LRTIs.

Pathogens	Detected by mNGS		Detected by CMTs		*P*-value
	All	mNGS positive	All	CMTs positive	
Matched lung cancer patietnts					
Bacteria	69	66 (95.7%)	62	14 (22.6%)^a^	<0.001
Fungi	50	48 (96.0%)	45	10 (22.2%)	<0.001
Matched non-lung cancer patietnts					
Bacteria	63	49 (77.8%)	61	25 (41.0%)^b^	<0.001
Fungi	36	26 (72.2%)	35	14 (40.0%)	0.006

^a^Included two BALF samples with positive mycobacterium tuberculosis DNA, ^b^Included one BALF sample with positive mycobacterium tuberculosis DNA and five BALF samples with positive mycobacterium tuberculosis X-pert, with the remaining cases confirmed by sputum culture.

### mNGS reveals LRT microbiome features in lung cancer patients

**Figure 3:**
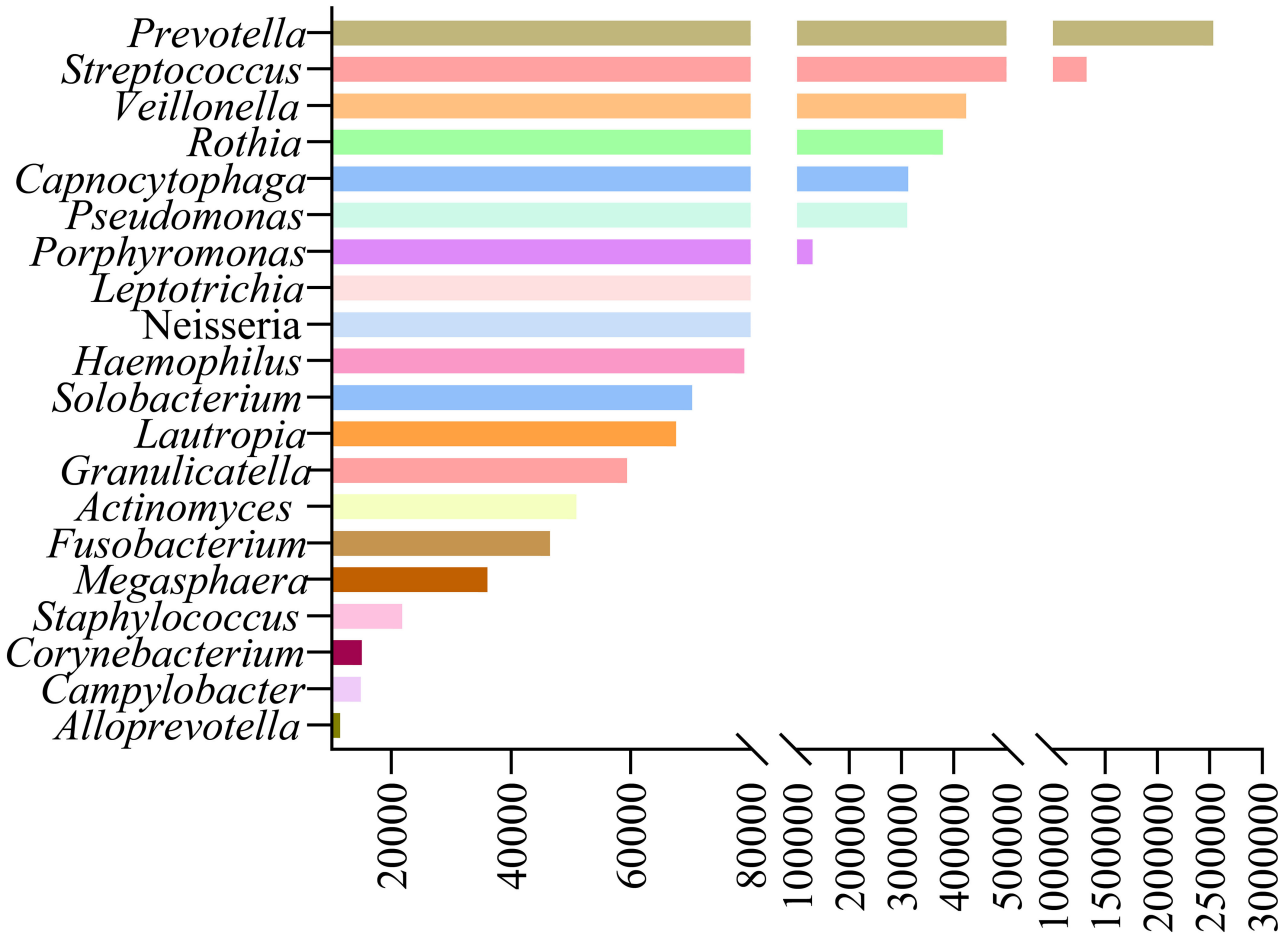
The predominant commensal/colonizing microbial genera in tumor-involved lobes of lung cancer patients.

**Figure 4:**
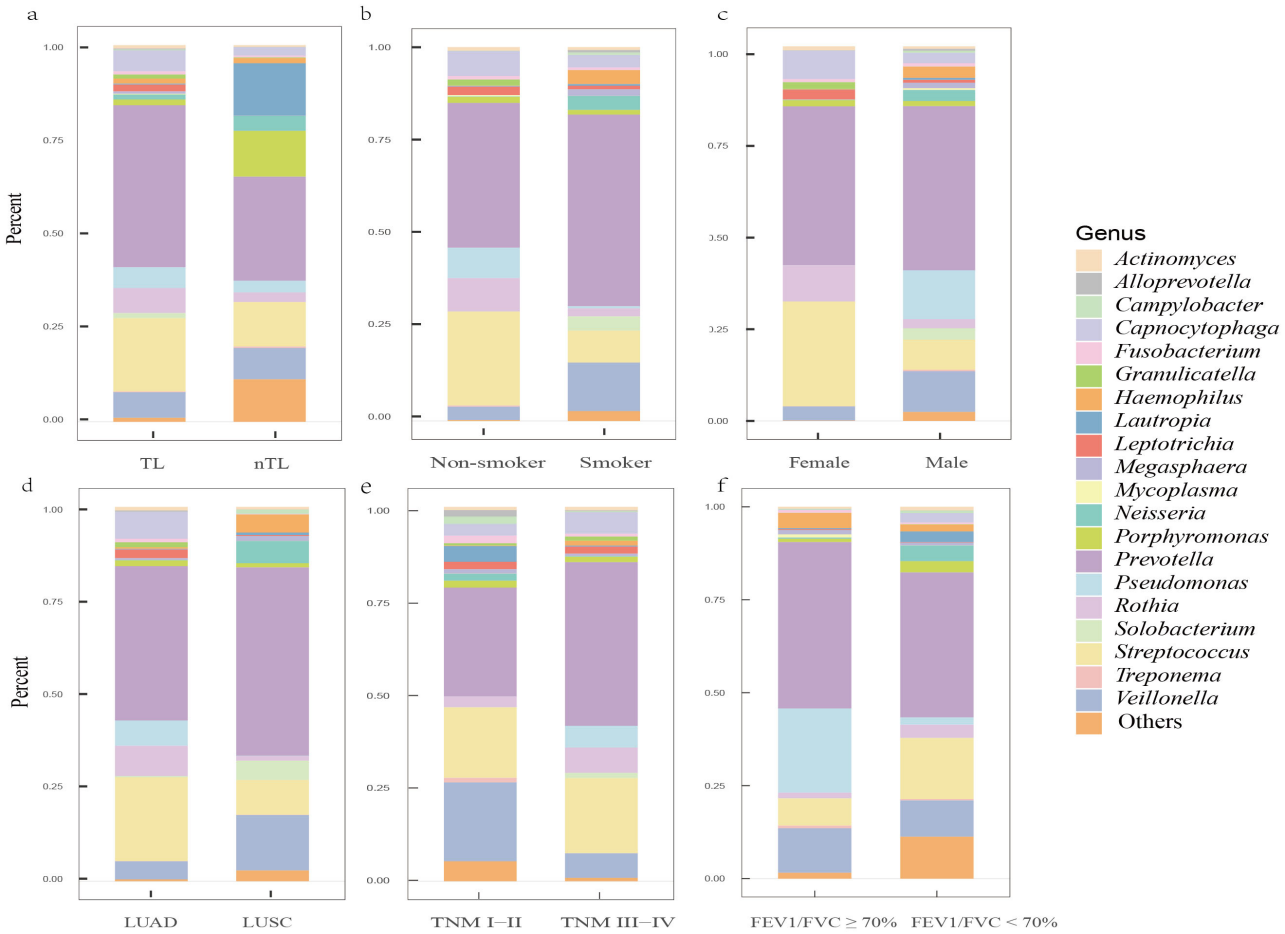
The predominant commensal/colonizing microbial genera in the LRT of lung cancer patients among different clinical subgroups. **(A)** Differences in microbial genera composition between tumor-involved lobe (TL) and non-tumor-involved lobe (nTL) in lung cancer patients; **(B)** Differences in microbial genera composition between non-smokers and smokers in TLs of lung cancer patients; **(C)** Differences in microbial genera composition between female and male in TLs of lung cancer patients; **(D)** Differences in microbial genera composition between lung adenocarcinoma (LUAD) and lung squamous cell carcinoma (LUSC) in TLs of lung cancer patients; **(E)** Differences in microbial genera composition between Tumor-Node-Metastasis (TNM) I-II and TNM III-IV in TLs of lung cancer patients; **(F)** Differences in microbial genera composition between Forced Expiratory Volume in 1 second/Forced Vital Capacity (FEV_1_/FVC) ≥ 70% and FEV_1_/FVC < 70% in TLs of lung cancer patients.

Our study analyzed background microbial sequence reads representing normal commensal/colonizing microbiota in the LRT from lung cancer patients’ mNGS data to characterize their LRT microbiome. mNGS analysis of BALF obtained from the tumor-involved lobe (TL) provides a characterization of the background microbiota, representing the tumor microenvironment in the LRT of lung cancer patients. Conversely, mNGS analysis of BALF from non-tumor-involved lobes (nTL) characterizes the non-tumor microbiota in these patients. The predominant bacterial genera in the TL of lung cancer patients were *Prevotella*, *Streptococcus*, *Veillonella*, *Rothia*, and *Capnocytophaga* and so on (**Figure 3**). Furthermore, this study additionally collected species-level reads of commensal microbiota between the two groups (**Supplementary Table 4**). Significant differences in microbial composition were observed between TL and nTL (**Figure 4A**). TL showed higher prevalence of *Prevotella*, *Streptococcus*, *Rothia*, and *Capnocytophaga* genera, while *Veillonella* was more abundant in nTL. Moreover, this study characterized the commensal/colonizing microbiota in BALF from TL of lung cancer patients and non-cancer controls to compare microbiome alterations (**Supplementary Tables 5**, **6**). The results showed significantly higher mapping read number of Prevotella, Streptococcus, Veillonella, Rothia, and Capnocytophaga in lung cancer patients compared to non-lung cancer patients. Microbial prevalence patterns varied among patient subgroups, stratified by smoking status, gender, tumor histology, TNM stage, and pulmonary function (**Figures 4B–F**). *Prevotella* and *Veillonella* were more prevalent in smokers, males, LUSC patients, and those with Forced Expiratory Volume in 1 second/Forced Vital Capacity (FEV1/FVC) ≥ 70%, but differed in TNM stages. *Prevotella* predominated in TNM stages III-IV, while *Veillonella* was more frequent in stages I-II. In contrast, *Streptococcus*, *Rothia*, and *Capnocytophaga* showed higher prevalence in non-smokers, females, LUAD patients, cases with FEV1/FVC < 70%, and those with TNM stages III-IV. These findings reveal distinct microbial prevalence patterns associated with smoking status, gender, histology, pulmonary function, and TNM stage, highlighting the complex interplay between the LRT microbiome and patient characteristics in lung cancer.

### GSVA

**Figure 5:**
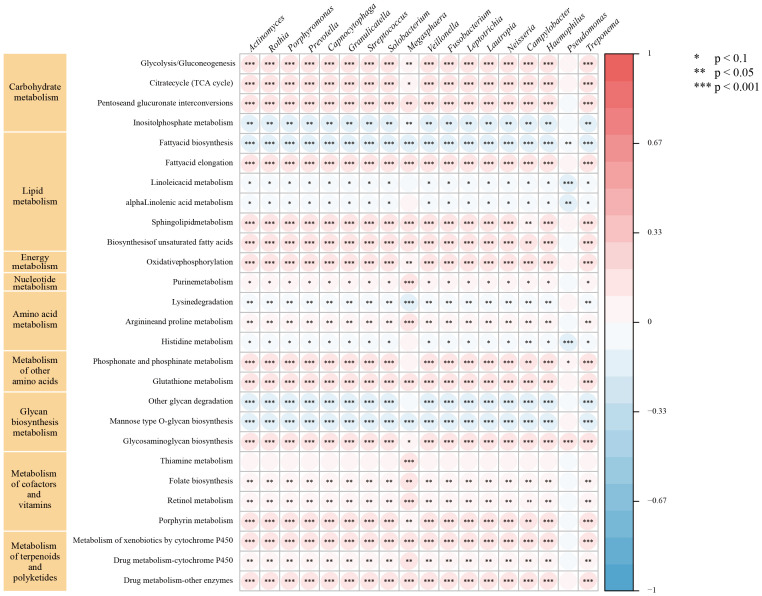
Relationship between intratumor microbe and metabolic pathway in TCGA-NSCLC patients. The symbol "*" indicates a p-value < 0.1, "**" indicates a p-value < 0.05, and "***" indicates a p-value < 0.001.

We further performed GSVA using gene sets from the KGEE database on transcriptomic data from TCGA-NSCLC samples (n=987). The results revealed that 18 microbes in lung cancer patients’ LRT significantly correlated (|r| > 0.2, P < 0.001) with 9 major metabolic pathways, primarily including carbohydrate, lipid, and energy metabolism, as well as metabolism of cofactors and vitamins (**Figure 5**). Among these, *Prevotella*, *Streptococcus*, *Veillonella*, *Rothia*, and *Capnocytophaga* exhibited negative correlations with 8 metabolic pathways, including inositol phosphate metabolism and fatty acid biosynthesis, among others. Conversely, these genera showed positive correlations with 19 metabolic pathways, encompassing the citrate cycle (TCA cycle) and glycolysis/gluconeogenesis, as well as additional related pathways. Additionally, we identified correlations between these common genera and pathways in Genetic Information Processing (DNA replication, nucleotide excision repair), Environmental Information Processing (PI3K-Akt, Notch, and Hippo signaling pathways), Cellular Processes (p53 signaling pathway), Organismal Systems (T and B cell receptor signaling pathways, IL−17 signaling pathway), and Human Diseases (Platinum drug resistance, Pathways in cancer, Small cell lung cancer) (**Supplementary Figures 1**–**5**). These findings contribute to the understanding of the unique biological role of microbiota in the lung tumor microenvironment.

## Discussion

Our study enrolled 110 lung cancer patients with suspected LRTIs, of whom 96 (87.3%) were diagnosed with infection based on mNGS, CMTs, and clinical data. Over half of the infections were polymicrobial, with bacteria being the most common, followed by fungi. In lung cancer patients with LRTIs, mNGS demonstrated a significantly higher detection rate for bacterial and fungal pathogens compared to CMTs. However, the difference was not significant when detecting viral infections, a finding consistent with results in non-lung cancer patients. Particularly in the case of sputum culture, mNGS demonstrated a higher pathogen detection rate in patients with LRTIs, regardless of their lung cancer status. Furthermore, Our study identified *Prevotella*, *Streptococcus*, *Veillonella*, *Rothia*, and *Capnocytophaga* as prevalent genera in TL of lung cancer patients, with distinct distribution patterns across clinical subgroups. Analysis revealed correlations between the metabolic activity of these genera and signaling pathways including PI3K-Akt, Hippo, and p53.

Despite growing interest in the unbiased, culture-independent diagnostic tool mNGS, its routine use for pathogen identification remains debated. Particularly for lung cancer patients, there is a lack of studies directly comparing the diagnostic performance of mNGS and CMTs in those patients with suspected LRTIs. Peng et al. (Peng et al., 2021) analyzed the diagnostic performance of mNGS compared to CMTs in immunocompromised patients, including 10% with solid tumors, and found no significant difference in overall diagnostic accuracy, particularly for fungal infections. Wang et al. (2024) reported superior performance of mNGS over CMT in detecting bacteria and fungi in severe pneumonia among cancer patients, which is consistent with our findings. However, only less than 35% of their cohort were lung cancer patients. In contrast, our study specifically evaluates the diagnostic performance of mNGS in lung cancer patients with LRTIs, highlighting its relevance in this particular population.

Lung cancer patients are prone to polymicrobial infections in the LRT, resulting in exacerbated respiratory symptoms. Understanding the distribution of pathogens in LRTIs among these patients is crucial for effective infection prevention and appropriate antibiotic use (Li et al., 2021). However, data on polymicrobial infections in cancer patients remain limited. Our study enhances understanding of LRTI epidemiology in lung cancer patients. Results show predominance of bacterial infections, primarily Gram-negative, followed by fungal infections, aligning with previous reports (Wang et al., 2023). For both lung cancer and non-lung cancer patients, mNGS outperformed CMTs in overall bacterial and fungal detection, particularly when compared with culture methods, and this is consistent with previous findings (Sun et al., 2022; Zhang et al., 2022) across different populations, which showed that mNGS pathogen detection rates (67%-80%) were significantly higher than those of traditional culture (19%-45%). These findings demonstrate the broad clinical applicability of mNGS, indicating that its diagnostic performance remains relatively stable across patients with different underlying diseases. White’s study (White et al., 2017) found *Aspergillus fumigatus* as the most common pathogen detected by mNGS in confirmed invasive aspergillosis pneumonia, with mNGS outperforming CMTs in diagnosis. This conclusion also applies to lung cancer patients in our study. Contrary to previous findings (Shi et al., 2022), our study revealed no significant difference in diagnostic yield between mNGS and CMTs for viral infections. Specifically, mNGS performance was comparable to CMV/EBV-DNA detection in plasma and whole blood, a well-established method for CMV/EBV infection in immunocompromised hosts (Nowalk and Green, 2016). All SARS-CoV-2 cases in our study were detected by pharyngeal swab testing, while this pathogen was not included in the mNGS detection panel. This finding suggests a potential need to expand the mNGS detection scope to encompass emerging pathogens in future studies. Our findings highlight the value of mNGS for diagnosing LRTIs, particularly for fastidious or slow-growing pathogens, in lung cancer patients. However, combining mNGS with conventional cultures may provide more comprehensive diagnostic information.

Previous studies (Yu et al., 2016; Liu et al., 2018; Narunsky-Haziza et al., 2022) have shown significant differences in LRT microbiome diversity between lung cancer patients and non-lung cancer patients. Specifically, lung cancer patients typically exhibit lower α-diversity in their microbiome. Additionally, some studies (Segal et al., 2013; Iksen et al., 2021) have found that lung cancer patients possess a richer supraglottic microbiome than non-lung cancer patients. They suggest that dysbiosis of the LRT microbiome may exacerbate pulmonary inflammation, as indicated by elevated neutrophil and lymphocyte levels in BALF (Segal et al., 2013). This dysbiosis might also regulate epithelial cell proliferation, differentiation, and invasion, thereby promoting the occurrence and progression of lung cancer (Iksen et al., 2021). Building on these findings, we used mNGS to characterize the LRT microbiome and explore potential carcinogenic mechanisms of enriched bacterial communities in lung cancer patients through GSVA. Previous studies (Dong et al., 2021; Sepich-Poore et al., 2021; Wang et al., 2022) have identified *Prevotella*, *Streptococcus*, *Veillonella*, *Rothia*, and *Capnocytophaga* as commonly enriched bacterial genera in the LRT of lung cancer patients, consistent with our findings. Among these genera, Capnocytophaga is considered a normal member of the oral microbiota in both animals and humans. Therefore, we further analyzed these populations at the species level (**Supplementary Table 6**). In the LRT of lung cancer patients, Capnocytophaga gingivalis was the most abundant species, followed by Capnocytophaga sputigena. Both species are found in the human oral cavity, and previous studies have identified Capnocytophaga sputigena as a pathogen in human infectious diseases (Kim et al., 2014; Liu et al., 2021b). Additionally, Capnocytophaga gingivalis has been shown to invade oral squamous cell carcinoma tissues and promote the invasion and metastasis of cancer cells (Zhu et al., 2024). Considering the potential influence of clinical features on LRT microbiota distribution in lung cancer patients (Ramírez-Labrada et al., 2020; Zheng et al., 2021; Kim et al., 2022), we stratified patients into subgroups for detailed microbial characterization. *Prevotella* and *Veillonella* were more associated with male, smoking, FEV1/FVC ≥ 70%, and squamous cell carcinoma patients. Notably, *Prevotella* was more prevalent in TNM stage III-IV, while *Veillonella* was more common in TNM stage I-II. Conversely, *Streptococcus*, *Rothia*, and *Capnocytophaga* were more prevalent in female, non-smoking, adenocarcinoma, TNM stage III-IV, and FEV1/FVC < 70% patients. These findings enhance our understanding of common microbial distribution across lung cancer subgroups. Some of our findings align with previous studies. Huang et al. (2019) found *Rothia* and *Capnocytophaga* enrichment in metastatic LUAD, and *Veillonella* in metastatic LUSC. Liu et al. (2018) noted higher *Streptococcus* abundance in lung cancer patients with emphysema versus those without. These findings underscore the potential association between LRT microbiota composition and clinical features in lung cancer patients. GSVA revealed positive correlations between these genera and diverse cancer metabolic pathways, including glycolysis and the TCA cycle. The Warburg effect demonstrates that cancer cells increase their glucose uptake and aerobic glycolysis to support tumor development (Nie et al., 2020). Recent evidence (Anderson et al., 2018) showed that some cancer cells, especially those with deregulated oncogenes and tumor suppressor expression, depend heavily on the TCA cycle for energy and biosynthesis. In addition, GSVA identified potential oncogenic pathways linked to these genera, including the PI3K-Akt pathway, known to be upregulated in early NSCLC and regulate cell proliferation, survival, differentiation, and invasion. Tsay et al. (2018) demonstrated that *Prevotella*, *Streptococcus*, and *Veillonella* promote PI3K pathway upregulation in airway epithelial cells, potentially contributing to lung carcinogenesis. Additionally, dysbiotic lung microbiota stimulated γδT cell proliferation and activation, inducing IL-17 production and promoting neutrophil infiltration and inflammation in the tumor microenvironment (Dong et al., 2021). Dysregulation of pathways such as Hippo signaling can induce lung cancer in model organisms (Harvey et al., 2013), while disruptions in P53 signaling pathways and platinum drug resistance enhance drug tolerance in lung cancer patients (Mo et al., 2022). These oncogenic and treatment-related pathways were also associated with these genera, but further experiments are needed to validate their role in lung cancer promotion via these pathways.

There were some limitations in our studies. Firstly, this retrospective study was limited by sample size. Future studies with larger cohorts are warranted to validate our findings. Secondly, prospective studies utilizing microbiome sequencing of BALF are recommended to comprehensively characterize the LRT microbiota in lung cancer patients. Lastly, further experimental studies are needed to elucidate the specific mechanisms by which microbes promote lung carcinogenesis.

## Conclusion

Our study evaluated mNGS performance in diagnosing suspected LRTIs in lung cancer patients and elucidated LRTI epidemiological patterns in this population. Furthermore, by analyzing background microbial sequence reads from mNGS data, we characterized the microbiome features of the LRT in lung cancer patients. GSVA was employed to explore potential carcinogenic mechanisms associated with identified genera. This study comprehensively assessed mNGS clinical utility in diagnosing LRTIs while investigating characteristics and biological significance of the lung cancer-associated microbiome.

## Data Availability

The data analyzed in this study is subject to the following licenses/restrictions: The datasets used and/or analyzed during the current study are available from the corresponding author on reasonable request. Requests to access these datasets should be directed to liu_y@hust.edu.cn and zhouqiongtj@hust.edu.cn.
