# Supplementary material for: Metagenomic next-generation sequencing for lung cancer low respiratory tract infections diagnosis and characterizing microbiome features
Source: Front Cell Infect Microbiol. 2025 Jan 23;14:1518199. doi: 10.3389/fcimb.2024.1518199 (PMC11799255; doi:10.3389/fcimb.2024.1518199)

Supplementary figure 1. Relationship between intratumor microbe and Genetic Information Processing in TCGA-NSCLC patients.





Supplementary figure 2. Relationship between intratumor microbe and Environmental Information Processing in TCGA-NSCLC patients.


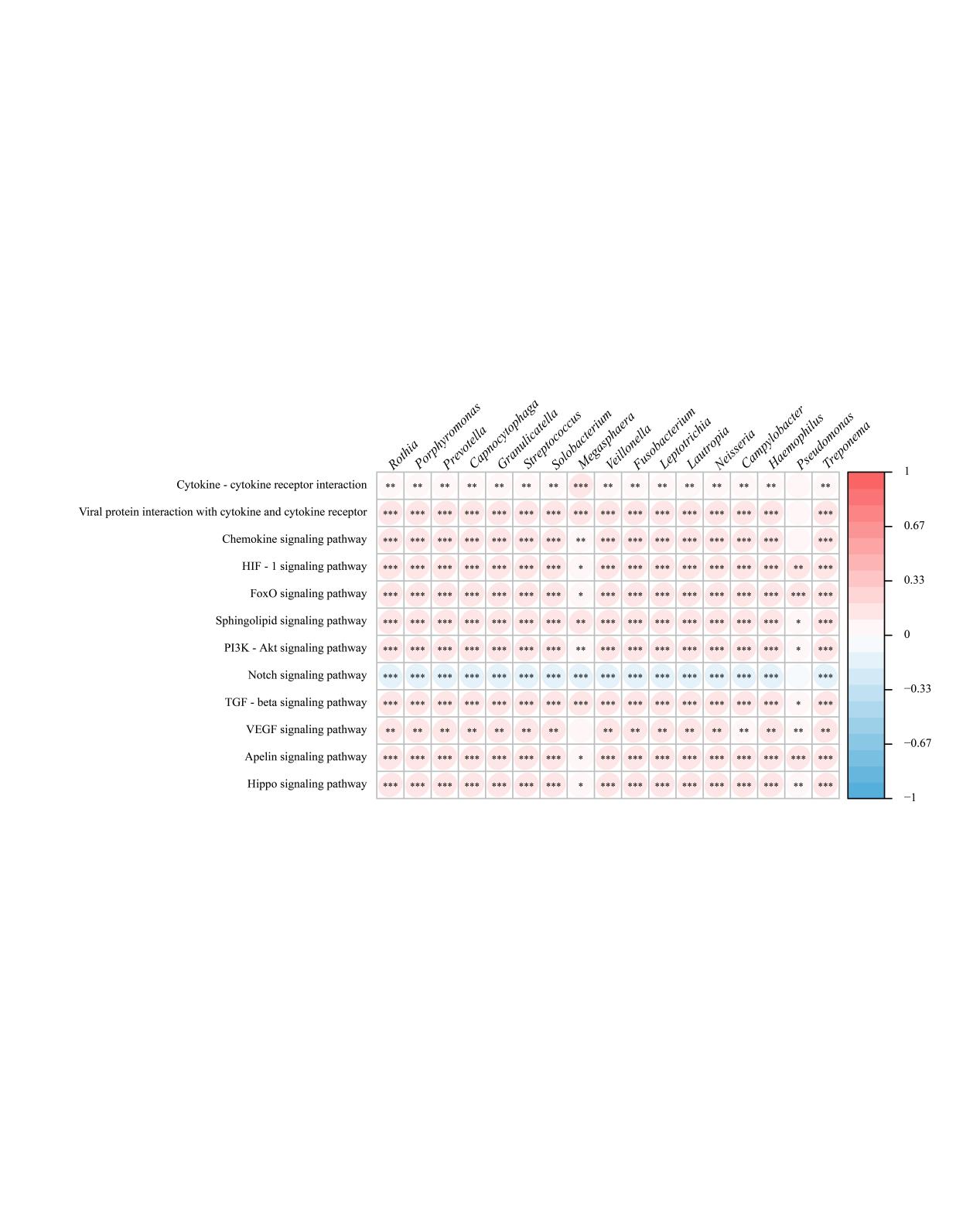


Supplementary figure 3. Relationship between intratumor microbe and Cellular Processes in TCGA-NSCLC patients.





Supplementary figure 4. Relationship between intratumor microbe and Organismal Systems in TCGA-NSCLC patients.


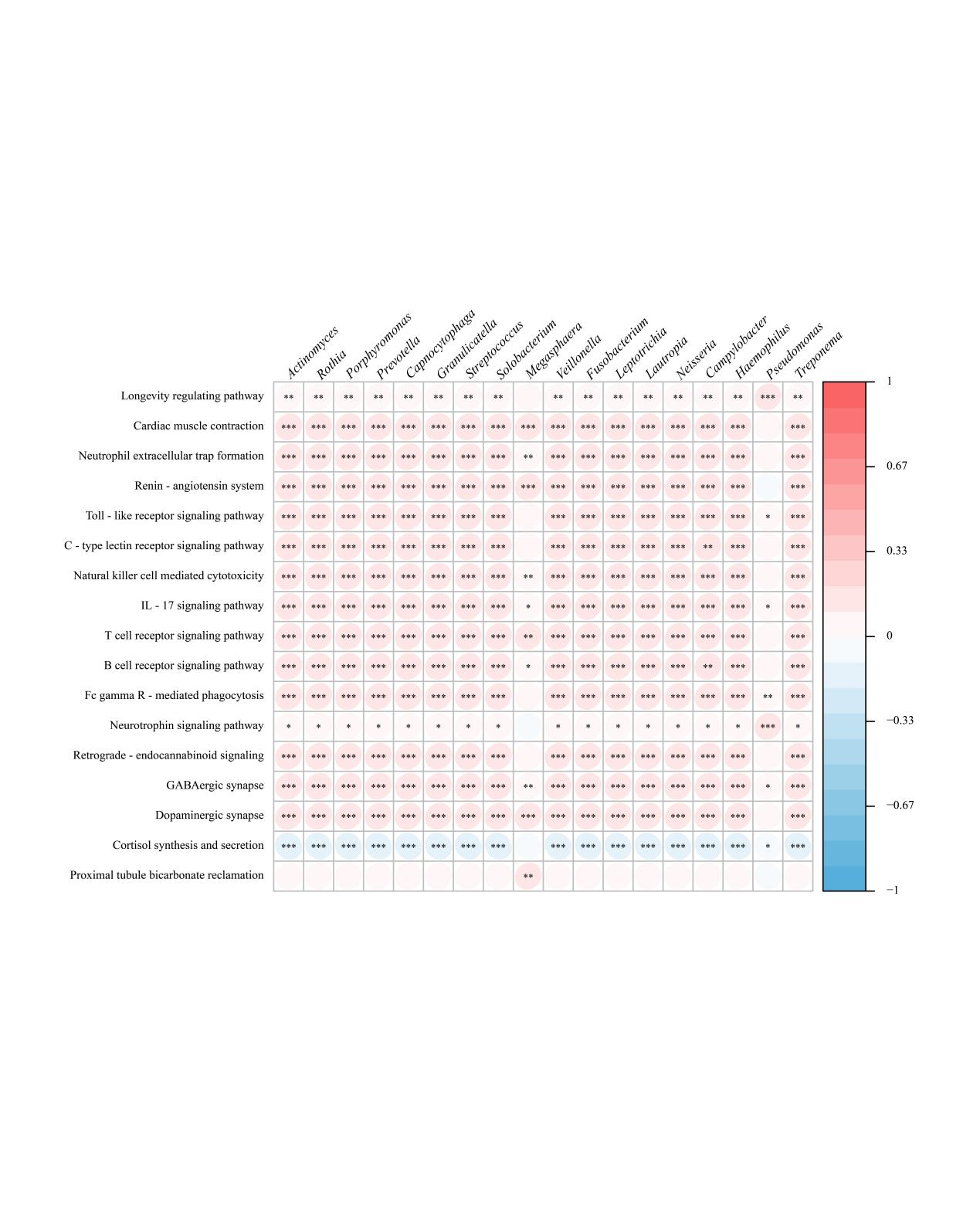


Supplementary figure 5. Relationship between intratumor microbe and Human Diseases in TCGA-NSCLC patients.


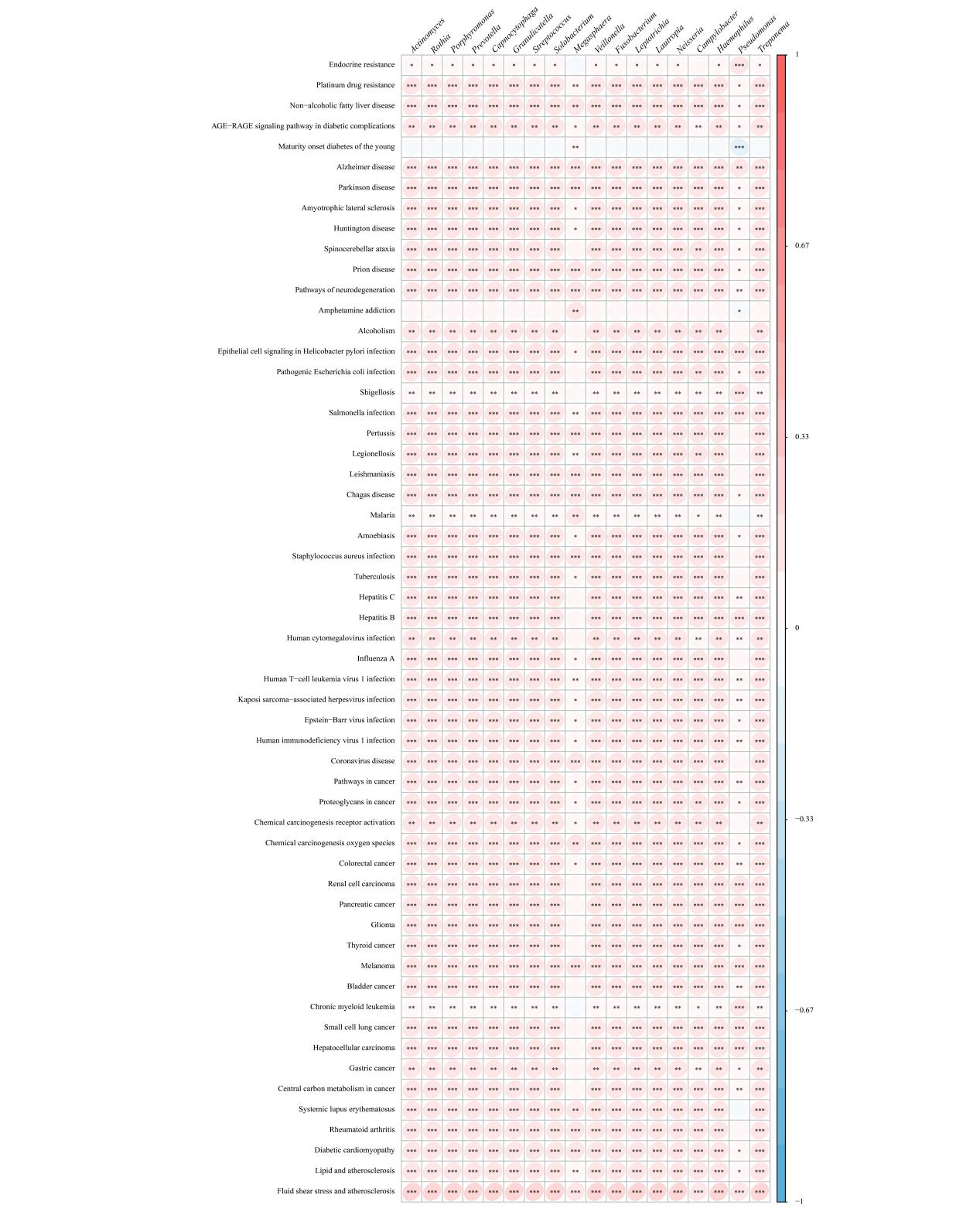

Supplement: Supplementary file 1 [file Table1.docx]
