# Supplementary material for: Metagenomic next-generation sequencing for lung cancer low respiratory tract infections diagnosis and characterizing microbiome features
Source: Front Cell Infect Microbiol. 2025 Jan 23;14:1518199. doi: 10.3389/fcimb.2024.1518199 (PMC11799255; doi:10.3389/fcimb.2024.1518199)
Supplement: Supplementary file 2 [file Table2.docx]

Supplementary table 1. Characteristics of lung cancer and non-lung cancer patients before and after PSM.

| Characteristics | Befor PSM | | | |  | After PSM | | | |
| --- | --- | --- | --- | --- | --- | --- | --- | --- | --- |
|  | Non-lung cancer patients(n=428) | lung cancer patients(n=110) | P value | SMD |  | Non-lung cancer patients(n=102) | lung cancer patients(n=102) | P value | SMD |
| Age | 58(50,68) | 62(58,69) | 0.001 | 0.582 |  | 62(56,70) | 62(57,69) | 0.908 | 0.000 |
| Gender |  |  | 0.001 |  |  |  |  | 1.000 |  |
| Male | 260(60.7%) | 87(79.1%) |  | 0.183 |  | 79(77.5%) | 80(78.4%) |  | 0.010 |
| Female | 168(39.3%) | 23(20.9%) |  | -0.183 |  | 23(22.5%) | 22(21.6%) |  | -0.010 |
| Application of antibiotics before mNGS | 374(87.4%) | 93(84.5%) | 0.531 | -0.028 |  | 91(89.2%) | 86(84.3%) | 0.409 | -0.049 |
| Application of glucocorticoid before mNGS | 68(15.9%) | 14(12.7%) | 0.500 | -0.032 |  | 9(8.8%) | 14(13.7%) | 0.376 | -0.049 |

* PSM, Propensity score matching; mNGS, metagenomic next-generation sequencing; SMD, standardized mean differences

Supplementary table 2. Evaluating the performance of mNGS and sputum culture in diagnosing LRTIs in lung cancer patients and non-lung cancer patients.

| Pathogens | detected by mNGS | |  | detected by sputum culture | |  | *P*-value |
| --- | --- | --- | --- | --- | --- | --- | --- |
|  | positive | negative |  | positive | negative |  |  |
| **Lung cancer patients** |  |  |  |  |  |  |  |
| infection | 70(83.3%) | 14(16.7%) |  | 29(34.5%) | 55(65.5%) |  | p<0.001 |
| non-infection | 3(27.3%) | 8(72.7%) |  | 1(9.1%) | 10(90.9%) |  | p=0.586 |
| **Matched Lung cancer patients** |  |  |  |  |  |  |  |
| infection | 64(82.1%) | 14(21.8) |  | 25(32.1%) | 53(67.9%) |  | p<0.001 |
| non-infection | 7(70.0%) | 3(30.0) |  | 1(10.0%) | 9(90.0%) |  | p=0.020 |
| **Matched non-lung cancer patients** |  |  |  |  |  |  |  |
| infection | 60(72.3%) | 23(27.7%) |  | 32(38.6%) | 51(61.4%) |  | p<0.001 |
| non-infection | 0(0.0%) | 1(100.0%) |  | 0(0.0%) | 1(100.0%) |  | p=1.000 |

*mNGS, metagenomic next-generation sequencing

| Supplementary table 3. Evaluating the performance of mNGS and quantification of viral DNA in diagnosing LRTIs in lung cancer patients and non-lung cancer patients. | | | | | | | |  |
| --- | --- | --- | --- | --- | --- | --- | --- | --- |
| Pathogens | detected by mNGS | |  | detected by quantification of viral DNA^a^ | |  | *P*-value | |
|  | all | positive |  | all | positive |  |  | |
| **Lung cancer patients** |  |  |  |  |  |  |  | |
| EBV | 7 | 7 |  | 5 | 3 |  | 0.152 | |
| CMV | 7 | 7 |  | 5 | 5 |  | 1.000 | |
| **Matched lung cancer patietnts** |  |  |  |  |  |  |  | |
| EBV | 5 | 5 |  | 4 | 4 |  | 1.000 | |
| CMV | 6 | 6 |  | 4 | 2 |  | 0.133 | |
| **Matched non-lung cancer patietnts** |  |  |  |  |  |  |  | |
| EBV | 6 | 4 |  | 3 | 3 |  | 0.500 | |
| CMV | 1 | 1 |  | 1 | 1 |  | 1.000 | |

* ^a^ included whole blood and plasma DNA; mNGS, metagenomic next-generation sequencing; DNA, deoxyribonucleic acid; EBV, Epstein-Barr Virus; CMV, Cytomegalovirus

Supplementary table 5. Mapping read numbers of predominant commensal/colonizing microbial genera in matched lung cancer and non-lung cancer patients.

| Genera | Mapping read numbers of colonizing bacteria in TL of matched lung cancer patients (n=57) | Mapping read numbers of colonizing bacteria in matched non-lung cancer patients (n=57) |
| --- | --- | --- |
| Prevotella | 2312652 | 1264696 |
| Streptococcus | 1042606 | 212592 |
| Veillonella | 355734 | 253898 |
| Rothia | 358237 | 84780 |
| Capnocytophaga | 303058 | 10156 |
| Pseudomonas | 297609 | 14 |
| Leptotrichia | 100415 | 28479 |
| Porphyromonas | 80222 | 14997 |
| Haemophilus | 73635 | 19875 |
| Neisseria | 70592 | 83491 |
| Solobacterium | 69815 | 0 |
| Granulicatella | 58574 | 135 |
| Actinomyces | 48077 | 40143 |
| Fusobacterium | 44711 | 33878 |
| Megasphaera | 32127 | 6278 |
| Campylobacter | 12658 | 2182 |
| Lautropia | 11804 | 6200 |
| Alloprevotella | 11435 | 1530 |
| Mycoplasma | 9853 | 68 |
| Treponema | 9560 | 1069 |
| Others | 49731 | 42112 |

*mNGS, metagenomic next-generation sequencing; BALF, bronchoalveolar lavage fluid; TL, tumor-involved lobe.
